# Malnutrition among HIV infected children under 5 years of age at the Laquintinie hospital Douala, Cameroon

**DOI:** 10.11604/pamj.2018.30.91.15832

**Published:** 2018-05-31

**Authors:** Calixte Ida Penda, Else Carole Eboumbou Moukoko, Nicolas Policarpe Nolla, Nadia Olivia Abomo Evindi, Paul Koki Ndombo

**Affiliations:** 1 Department of Clinical Sciences, Faculty of Medicine and Pharmaceutical Sciences, University of Douala, Cameroon; 2 HIV Care and Treatment Centre, Laquintinie Hospital of Douala, Cameroon; 3 Department of Biological Sciences, Faculty of Medicine and Pharmaceutical Sciences, University of Douala, Cameroon; 4 Department of Biochemistry, Faculty of Science, University of Douala, Cameroon; 5 Department of Pediatrics, Faculty of Biomedical Sciences, University of Yaoundé I, Cameroon

**Keywords:** Malnutrition, HIV infected children, Douala, Cameroon

## Abstract

**Introduction:**

The aim of this study was to assess the prevalence of malnutrition among HIV infected children under five years of age followed up at the Laquintinie Hospital Douala (LHD).

**Methods:**

Medical records of children aged 13 days-59 months enrolled at initiation of antiretroviral treatment in the Day Care Unit/LHD, were reviewed for a period of 14 years (from 2002 to 2015). We used standard Z-scores, with cut-off point of <-2 SD to define low height-for-age (HAZ), low weight-for-height (WHZ) and low weight-for-age (WAZ). Factors associated with malnutrition were assessed according to World Health Organization (WHO) criteria.

**Results:**

Overall, 217 medical records were included and 52.5% were records of boys. The median weight, height and age of the children was 9.5 kg (range: 2.5-20), 76 cm (range: 46- 117) and 22 months (range: 0.03-59), respectively. The overall prevalence of malnutrition among HIV-infected children was 68.7%; 63.6% were stunted (HAZ<-2), 37.8% were underweight (WAZ<-2) and 18.4 % were wasted (WHZ<-2). Severe and advanced immunological stages of HIV according to WHO were found in 42.4%, (39/92) and 17.4%, (16/92) of children respectively, and most of them (21.7%) were aged 12-36 months. The overall prevalence of anemia, oropharyngeal candidiasis and pulmonary tuberculosis were 34.6%, 12% and 8.8%, respectively. Oropharyngeal candidiasis was a risk factor independently associated with severe underweight and wasting (OR = 4.9, 95% CI: 1.8-13.5, p = 0.002) and (OR = 5.1, 95% CI: 1.5-17.1, p = 0.007).

**Conclusion:**

HIV infection negatively affects the nutritional status of children under five years of age. Early detection of malnutrition is necessary and adequate nutrition should be integrated into the management of pediatric HIV.

## Introduction

Sub-Saharan Africa is one of the most affected regions by malnutrition, with 31.2% of children under 5 years of age suffering from stunting, 5.2% of overweight and 7.4% of wasting. Severe forms occur in 2.2% of the population according to a report in 2015 [1]. In Cameroon, 31.7% of children under 5 years of age suffer from stunting, 5.2% from wasting and 14.8% from underweight [2]. Chiabi et al (2012) and Sobze et al (2014) in Cameroon reported respectively that 66.7% and 31.5% of children under 5 years of age were malnourished [3,4]. Malnutrition is an important cause of child morbidity and mortality in Cameroon. Every year, malnutrition accounts for more than one third of all deaths among children under five years in resource-limited-settings [5]. It increases susceptibility to infections and predisposes to poor physical and cognitive development [6,7]. Among children with severe malnutrition, the risk of death is three times higher in HIV-infected children compared to those who are not infected [8]. Human immunodeficiency virus (HIV) infection is also a major public health problem in children in Sub-Saharan Africa. In 2015 about 1.8 million children under 15 years of age were infected with HIV worldwide, with more than 90% of them found in sub-Saharan Africa [9]. About 52 million children under five years of age suffer from wasting, while 155 million are affected by stunting and 41 million are overweight [1]. Malnutrition is a major problem especially for HIV-infected children. There is a vicious circle between HIV infection and malnutrition, this vicious circle contributes in depressing the child's immune system. Immunodepression combined with HIV infection and malnutrition is a determinant of poor prognosis for child survival even with antiretroviral therapy (ART). Early identification of malnutrition is necessary to undertake preventive measures and appropriate therapeutic strategies. This study was conducted to determine the prevalence of malnutrition among HIV-infected children under 5 years of age at initiation of ART at the HIV Care and Treatment Centre (HIV/CTC) of the Laquintinie Hospital of Douala (LHD).

## Methods

**Study setting**: The study was carried out in the Littoral region, precisely in the HIV/CTC of the LHD. The LHD is one of the national reference hospital, a specialized center for the management of malnutrition in children and a center of excellence for pediatric HIV care.

**Study design**: A retrospective study was carried out in a cohort of HIV-infected children, aged 0-5 years at initiation of ART and regularly followed at the HIV/CTC of the LHD from January 2002 to December 2015, for a period of 14 years. Children's medical records were included consecutively in the study. Medical records of children with missing data and chronic diseases such as sickle cell disease, renal failure and heart failure and non-HIV-related tumors that can influence growth, were excluded.

**Ethical aspects**: The Institutional Review Board of the Faculty of Health Sciences of the University of Buea in Cameroon approved the study N°2013/134/UB/FHS/IRB and administrative authorization for research N°1098/AR/MINSANTE/DHL/CM/DM was obtained from the LHD. Throughout the study, nurses were trained and all the staff involved in the follow up of HIV infected children was educated.

### Overall study design and data collection

*Z-scores calculation*: all the medical records of HIV infected children appropriately followed up were collected at the HIV/CTC. Information about nutritional status was obtained using the WHO Anthro and AnthroPlus software's for health data for assessing growth and development (length/height) of the children. The weight was measured before every consultation. We used Salter type weighing scales with a range of 0-25 kg with an accuracy of 100 g. To determine the child's length, children younger than 2 years laid on a flat surface. The measurement of height for children aged 2 years or more was taken while standing (heels against the wall, without shoes). The Z-scores were calculated using the anthropometric measurements according to the age and the sex of each child.

*Classification of malnutrition*: we classified malnutrition into three groups according to standard deviations of Z-score described in WHO classification [10]. Stunting or chronic malnutrition *(child with small height for age)* is due to multiple deficiencies and repeated infectious episodes in-utero or in early childhood. The child is below the height curve for his age. Stunting is classified as moderate when the height-for-age Z-score is between -2 SD and -3 SD and severe when below -3 SD compared to the reference population. Wasting or acute malnutrition *(child with low weight for height)* is characterize by a recent weight loss due to deficiency or acute infection, a reduced body mass, impaired vital functions and a greater risk of death. Wasting is classified moderate when the Z-score weight for height is between -2 SD and -3 SD and severe when below -3 SD compared to the reference population. Underweight *(child with low weight for age)* describes a thin child but, this definition does not allow discriminating acute malnutrition from chronic malnutrition. This indicator allows easy tracking of the nutritional status outcome of a child, as moderate when the weight for age Z-score is between -2 SD and -3SD and severe when below -3 SD.

*Clinical definition and biological data*: edema: pretibial, bilateral, facial or generalized swelling at admission. Immunodeficiency was defined according to WHO recommendations [11]: no immunodeficiency for children whose CD4% was more than 35%, 30% and 25% for the age groups <11 months, 12-35 months and 36-59 months respectively. Moderate immunodeficiency for children with CD4% ranged between 30-35%, 25-30% and 20-25%, for the age groups <11 months, 12-35 months and 36-59 months respectively. Advanced immunodeficiency for children with CD4% ranged between 25-29%, 20-24% and 15-19%, for age groups <11 months, 12-35 months and 36-59 months respectively. Severe immunodeficiency for children with CD4% was <25%, 20% and 15%, for the age groups <11 months, 12-35 months and 36-59 months respectively. Anemia according to WHO criteria was defined by a hemoglobin level of less than 11.0 g/dl [12]. Oropharyngeal candidiasis, fungal infection was defined as the presence of white plaques, creamy-looking patches in the mouth and pharynx on the surface of the tongue, oral mucosa or pharynx outside the neonatal period. The diagnosis of pulmonary tuberculosis was made if one or more of the following conditions were met: clinical signs of tuberculosis (notion of contact, fever, cough, weight loss); signs consistent with tuberculosis at chest X-ray; isolation of *Mycobacterium tuberculosis* in the gastric fluid specimen obtained by gastric lavage; positive tuberculin skin test (induration ≥5 mm).

**Statistical analysis**: Anthropometric data was analyzed using SPSS version 16 and WHO Anthro version 3.2.2 statistical software. Logistic regression analysis was used to identify factors associated with the variables characterizing malnutrition and the clinical characteristics. Adjusted Odds Ratios (OR) as well as their 95% Confidence Intervals (CI) were computed. The variables of interest were used in a univariate logistic model and selected variables were analyzed using a multivariate logistic model [13]. P-values <0.05 were considered statistically significant.

## Results

**Socio-demographic characteristics of studied population**: Table 1 describes the information about the study population. Overall, 446 files were retrieved from which 217 files of HIV-infected children were included. Boys accounted for 52.5% of patients giving a male to female sex ratio of 1/1.1. The mean age was 26 months (SD=18). The frequency of children aged 36 to 59 months (32.3%) was twice higher than those in the age group between 6 and 12 months (15.2%). Children aged 12 to 24 months (25.8%) were twice as much as the children aged 0 to 6 months (12.4%). Up to 81.7% and 89.7% of the children had their mothers or fathers still alive respectively. Only 18.3% of children were orphaned from mother, 10.3% from father and 10.2% from both parents. About 58.1% of the mothers were single and 41.9% were married. More than half of mothers (65.6%) had secondary level of education and 46.2% were unemployed.

**Table 1:** Socio-demographic characteristics of the study population

Variables	Numbers	Percent
**Age group of children (months), N=217**		
[0 - 6]	27	12.4
[6 - 12]	33	15.2
[12 - 24]	56	25.8
[24 - 36]	31	14.3
[36 - 60]	70	32.3
**Sex of children, N=217**		
Boys	114	52.5
Girls	103	47.5
**Mothers, N=213^a^**		
Mothers still alive	174	81.7
Dead mothers	39	18.3
**Fathers, N=194^b^**		
Fathers still alive	174	89.7
Dead fathers	20	10.3
**Dead parents, N=59**		
Both parents dead	6	10.2
**Mother’s marital status, N=167^c^**		
Married^*^	70	41.9
Single^$^	97	58.1
**Mother’s education level, N=157^d^**		
No Formal education	1	0.6
Primary	29	18.5
Secondary	103	65.6
Higher	24	15.3
**Mother’s employment status, N=169^e^**		
Civil servant	16	9.5
Private sector	15	8.9
Informal sector	53	31.4
Trader	7	4.1
Unemployed	78	46.2
**Father’s employment status, N=159^f^**		
Civil servant	14	8.8
Private sector	41	25.8
Informal sector	77	48.4
Trader	12	7.5
Unemployed	15	9.5

a No available data for 4 mothers

b No available data for 23 fathers

c No available data for 7 mothers

* Married or concubines

$ Divorced/Separated or Widowed

d No available data for 17 mothers

e No available data for 5 mothers

f No available data for 15 fathers

**Immunological status of HIV-infected children**: Of the 217 files included in the study, only 42.4% had CD4 counts. The CD4% value ranged from 7.6% to 68.4% for children <11 months, between 0.4% to 67.4% for those 12-36 months and between 0.3% to 53.8% for those 36-59 months. Most of these children had severe immunodeficiency (42.4%, 39/92) or advanced immunodeficiency (17.4%, 16/92) and most of them (21.7%) were aged 12-36 months.

**Nutritional status and gender**: The overall prevalence of malnutrition among HIV-infected children at admission to LHD was 68.7%, and among them 21.7% were moderate and 47% were severe. In the case of stunting, severe forms represented 43.8%, mean Z-scores (HAZ <-3) = -4.87 (SD = 1.7); p= 0.81) and moderate forms made up 19.8% (Mean Z score (-3 ≤HAZ < -2) = -2.48 (SD=0.3); p=0.94); Severe underweight (24%, mean Z-scores (WAZ <-3) = -4.31 (SD = 1.2), p = 0.26) and severe wasting (10.6%, mean Z-scores (WHZ <-3) = -4.04 (SD = 0.8), p = 0.07). No significant difference was found by comparing the moderate and severe forms with the reference as described in Table 2. More than half children with severe malnutrition suffered from severe stunting. No difference was observed between girls and boys in the prevalence of individual forms of malnutrition.

**Table 2:** Nutritional status of children by sex

Variables	Boys (n=114)	Girls (n=103)	Total N (%)	OR (95% CI)	P value
**Stunting (n=217)**					
Normal	41 (18.9)	38 (17.5)	79 (36.4)	1	-
Moderate	22 (10.1)	21 (9.7)	43 (19.8)	0.97 (0.46 - 2.04)	0.94
Severe	51 (23.5)	44 (20.3)	95 (43.8)	1.07 (0.59 - 1.95)	0.81
**Underweight (n=217)**					
Normal	68 (31.3)	67 (30.9)	135 (62.2)	1	-
Moderate	15 (6.9)	15 (6.9)	30 (13.8)	0.98 (0.45 - 2.17)	0.97
Severe	31 (14.3)	21 (9.7)	52 (24)	1.45 (0.76 - 2.78)	0.26
**Wasting (n=217)**					
Normal	86 (39.6)	91 (42)	177 (81.6)	1	-
Moderate	12 (5.5)	5 (2.3)	17 (7.8)	2,51 (0.85 - 7.42)	0.09
Severe	16 (7.4)	7 (3.2)	23 (10.6)	2.39 (0.94 - 6.10)	0.07

Data are number (N) and proportion (%) of variable, unless otherwise indicated; OR: Odds Ratio; CI: Confidence Interval;

**Nutritional status and age groups**: Children were divided into 5 groups according to age groups and the age group of 0-6 months was considered as the reference. Stunting was the most common form of malnutrition encountered in HIV infected children. The risk of stunting was significantly higher (12-fold) in 6-12 months old children compared with the age group 0-6 months (baseline) (OR = 12, 95% CI: 1.9- 76, p = 0.008) as shown in Table 3. In different proportions, the same trend was observed in children aged 12-24 months (OR = 11, 95% CI: 1.2 - 95, p = 0.03) and 24-36 months (OR = 9, 95% CI: 1.5-52, p = 0.01). For underweight and wasting, there was no significant difference between age groups.

**Table 3:** Severity of nutritional status by age groups

Age groups (months)	Stunting		Underweight		Wasting	
	OR (95% CI)	P value	OR (95% CI)	P value	OR (95% CI)	P value
[0 - 6] ^*^ (N= 27)	1	-	1	-	1	-
[6 - 12] (N= 33)	12 (1.9 - 76)	0.008	1.4 (0.2 - 11)	0.72	1.7 (0.2 - 18)	0.64
[12 - 24] (N= 56)	11 (1.2 - 95)	0.03	1.1 (0.2 - 7.9)	0.91	0.89 (0.1 - 8.1)	0.85
[24 - 36] (N= 31)	9 (1.5 - 52)	0.01	1.5 (0.2 - 13)	0.71	2 (0.2 - 27)	0.6
[36 - 59] (N= 70)	4.6 (1 - 20.9)	0.05	0.88 (0.1 - 6.5)	0.91	2 (0.2 - 27)	0.6

Data are number (N) and proportion (%) of variable

* reference age groups; OR: Odds Ratio; CI: Confidence Interval

**Nutritional status and opportunistic infections**: Overall, 26 (12%) HIV infected children had pulmonary TB, 19 (8.8%) had oropharyngeal candidiasis and 75 (34.6%) had anemia (Table 4). Among children with stunting, 23.5% were anemic and pulmonary tuberculosis and oropharyngeal candidiasis were found in 6% and 7.4% of children respectively. Children with severe stunting had the most opportunistic diseases, but there was no significant difference between stunted children and those with normal growth. Oropharyngeal candidiasis was significantly associated with severe underweight (OR = 4.9, 95% CI 1.8-13.5, p = 0.002), moderate wasting (OR = 7.7, 95% CI 2.2-26.7, p = 0.001) and severe wasting (OR = 5.1, 95% CI 1.5-17.1, p = 0.007). Pulmonary tuberculosis and anemia were present in HIV-infected children but were not factors contributing significantly to malnutrition in any form.

**Table 4:** Risk factors associated with malnutrition in HIV-infected children

Variables	Clinical features					
	Oropharyngeal candidiasis		Pulmonary Tuberculosis		Anaemia	
	OR (95% CI)	P value	OR (95% CI)	P value	OR (95% CI)	P value
**Stunting**						
Normal	1	-	1	-	1	-
Moderate	1.6 (0.5 - 5.6)	0.46	0.7 (0.2 - 2.4)	0.58	0.9 (0.4 - 2)	0.77
Severe	1.1 (0.4 - 3.4)	0.84	1 (0.4 - 2.4)	0.99	1.6 (0.8 - 3)	0.14
**Underweight**						
Normal	1	-	1	-	1	-
Moderate	0.6 (0.07 - 5.3)	0.67	0.2 (0.02- 1.4)	0.1	0.7 (0.3 -1.7)	0.43
Severe	4.9 (1.8 - 13.5)	0.002	0.4 (0.1 - 1.4)	0.2	1.3 (0.7 -2.5)	0.42
**Wasting**						
Normal	1	-	1	-	1	-
Moderate	7.7 (2.2 - 26.7)	0.001	0.4 (0.05 - 3.1)	0.4	2.2 (0.8 -6.1)	0.12
Severe	5.1 (1.5 - 17.1)	0.007	0.3 (0.04 - 2.2)	0.2	0.8 (0.3 -2.2)	0.77

## Discussion

This study provides information on malnutrition in HIV- infected children under five years of age followed up at the HIV/CTC of the LHD. Among children, 63.6%, 18.4% and 37.8% presented the stunting, wasting and underweight respectively. Globally, a there is a high prevalence (68.7%) of malnutrition among HIV- infected children at initiation of ART. This prevalence is in line with results obtained by Chiabi et al. (66.7%) in Yaoundé, Cameroon [3], less than that found by Poda et al (77%) among HIV-infected children in BOBO-Dioulasso, Burkina Faso [14], but higher than that found by Mwadianvita et al (60.2%) in the Democratic Republic of Congo [15]. These differences can be explained by the varying prevalence of malnutrition among HIV-infected children in different countries [4, 14,15] and by the different sample sizes in different studies (39,164 and 83 children respectively). In addition, it has been shown that HIV infection deteriorates the nutritional status of patients, even when ART is initiated [16]. Stunting (63.6%), was the most common type of malnutrition among HIV-infected children at initiation of ART. Among them 43.8% had severe forms. This is like that reported age in Central and Western Africa (Chad, Cameroon, Mali, Ivory Coast, Togo, Benin) [17] but higher than that reported by studies in Nigeria and southern India [18,19]. A previous study confirmed that stunting and nutritional status deterioration in children were associated with HIV infection [20]. The late diagnosis of HIV infection, with resultant significant immunodeficiency, the duration of the disease, and inadequate nutrition are factors associated with chronic malnutrition [15, 19-21]. Malnutrition and HIV have common biological, immunological and socio-economic consequences. These two conditions interact and create a vicious circle. Malnutrition impairs the immune system, especially in children [21-23]. In our study, 18.4% of the children presented wasting and among them 10.6% with severe forms. This prevalence is lower than that found by Mwadianvita et al in Lubumbashi, Democratic Republic of Congo and Anigilaje and Olutala in Makurdi, Nigeria who had prevalence of 20.5% and 33.5% respectively [15,18]. In central and west Africa and in southern India, the prevalence were 16% and 14% respectively [17,19].

The prevalence of underweight was 37.8%, of which 24% had the severe form. This prevalence was lower than that reported in HIV-infected children in southern India with 63% of cases [16], but higher than 12.1% found in Makurdi in Nigeria [18]. The prevalence of stunting, underweight and wasting varied according to the age groups. The age groups 6-12 months and 24-36 months had a high prevalence of severe underweight, severe wasting and severe stunting. These two age groups are vulnerable periods for children and correspond to the introduction of complementary feeding and dietary diversification while older children have an increasing frequency of common childhood diseases related to the gradual decline of their immunity. Children in the age group 0-6 months were the least affected with various forms of malnutrition, probably due to exclusive breastfeeding that is a protective factor against malnutrition during the first six months of life. Thus, the age of the child may be a factor of vulnerability to malnutrition because of the high nutritional needs for its growth and development. Most HIV- infected children had severe immunodeficiency as found by Ogunbosi et al in Nigeria with a slightly higher prevalence (56.8 % vs 42.1 %) in our population [24]. The presence of opportunistic infections such as pulmonary tuberculosis, oropharyngeal candidiasis and anemia is frequent and could explain the profound alteration of the nutritional status of HIV-infected children as revealed by Trehan et al, in Sub-Saharan Africa [25]. Anemia, a possible consequence of malnutrition, is also a specific complication of HIV infection that can cause stunting [26]. On contrary, Shet et al, reported that anemia is significantly associated with underweight and stunting, but not wasting [27]. People with iron deficiency anemia are at higher risk of infection and this can contribute to exacerbate the growth disorders and child morbidity and mortality. Oropharyngeal candidiasis was associated with severe underweight and severe wasting, probably due to difficult food intake and has a negative impact on the nutritional status of children [25]. HIV infection may also indirectly affect the nutritional status of children when it impacts on the social environment of children [28]. This study showed that 46.2% of mothers and 9.5% of fathers were unemployed, 31.4% of mothers and 48.4% of fathers were in the informal sector. This economic situation of parents may therefore be an important factor affecting malnutrition in children [29,30] because the risk of malnutrition is 8 times higher among children living in households with insufficient food budgets [31].

## Conclusion

Malnutrition is common among HIV-infected children. Oropharyngeal candidiasis is a deleterious factor among HIV-infected children with malnutrition in our study. Early detection of HIV and malnutrition as well as concomitant adequate nutrition should improve the care of these children.

### What is known about this topic

- Significant vulnerability related to the coexistence of 2 pathologies;
- Late management due to late detection related to lack of use of growth charts in children;
- Poor consideration of child nutrition in the implementation of pediatric HIV care programs.

### What this study adds

- Malnutrition was higher among HIV-infected children in our study. Oropharyngeal candidiasis, pulmonary tuberculosis and anemia are frequent and poor socioeconomic situation of parents has a deleterious effect;
- The implementation of systematic nutritional status check during routine consultations and pediatric antiretroviral therapy initiation and follow-ups would help to reduce child mortality;
- This study recommends the inclusion of a systematic assessment of nutritional status in pediatric HIV care programs and organizational strategies to improve care for HIV-infected children.
